# Supplementary material for: Uncertainty-driven regulation of learning and exploration in adolescents: A computational account
Source: PLoS Comput Biol. 2020 Sep 30;16(9):e1008276. doi: 10.1371/journal.pcbi.1008276 (PMC7549782; doi:10.1371/journal.pcbi.1008276)
Supplement: S1 Text — (DOCX) [file pcbi.1008276.s001.docx]

**Supplementary Text 1. Deviations from preregistration**

**Regression analyses (estimation task)**

Consistent with our preregistration, we tested the effects of trial, noise level and age group on certainty and learning rate. However, we deviated from our preregistration in *how* we modeled the effects of trial. According to our preregistration, we would fit power functions to each participant’s timecourse of certainty ratings and learning rates—separately for the two noise conditions—and then perform regression analyses on the estimated power-function parameters. After preregistering, however, we realized that this power-function approach was too specific for our research questions, and that a more flexible and parsimonious approach would be to model the linear and quadratic effects of trial (which also allowed the use of multilevel models).

**Mediation analysis (estimation task)**

We preregistered a related but different analysis, namely a regression of learning rate on certainty and age group. However, as both certainty and learning rate were strongly related to trial number, we reasoned that it would be more informative to control for trial in this analysis. Therefore, we used a multilevel moderated mediation analysis that simultaneously tests (i) whether trial-to-trial changes in certainty are predictive of learning rate when controlling for trial number (path *b* of the mediation model); (ii) whether certainty mediates the relationship between trial number and learning rate; and (iii) whether each of these effects differ between age groups.

**Exclusion of outlier learning rates in the behavioural analyses of learning rate (estimation task)**

We did not preregister the exclusion of trials on which the estimated learning rate exceeded the 99^th^ percentile or was lower than the 1^st^ percentile. These most extreme learning-rate estimates likely resulted from occasional typing errors (e.g., when a participant accidentally typed ‘3’ instead of ‘83’)—as verified by inspection of the estimation data—something we did not anticipate when preregistering. Note that this exclusion criterion is usual in studies where learning rates are directly estimated from single-trial estimation data (Vaghi et al., 2017).

**Analyses of learning rate as a function of age within the adolescent group**

We did not preregister the analysis reported in the “Learning rate decreases over the course of early adolescence” section; hence this analysis should be considered exploratory.

**Computational models**

We preregistered the standard reinforcement learning and Kalman filter models, but not the asymmetric reinforcement learning and reinforcement learning/Pearce-Hall hybrid models. We added these latter two models to examine potential alternative explanations for the observed age-related differences in learning rate and choice behaviour, as suggested by the reviewers.

**Analyses of the choice task**

The reported analyses of the choice data were not preregistered, because we initially intended to report the results from this task separately, focusing on a different question (see http://aspredicted.org/blind.php?x=av4td4). However, after preregistering, we realized that the estimation and choice tasks yield complementary information about uncertainty-driven changes in reinforcement learning; hence could provide converging evidence regarding developmental changes. Therefore, we decided to analyze the choice data in a similar way as the estimation data, and report both sets of results together.
