# Supplementary material for: Uncertainty-driven regulation of learning and exploration in adolescents: A computational account
Source: PLoS Comput Biol. 2020 Sep 30;16(9):e1008276. doi: 10.1371/journal.pcbi.1008276 (PMC7549782; doi:10.1371/journal.pcbi.1008276)
Supplement: S2 Text — (DOCX) [file pcbi.1008276.s002.docx]

**Supplementary Text 2. Optimal adaptation of learning rate in noisy but static environments**

When outcomes are drawn from a static Gaussian distribution—as in our experimental tasks—the best estimate of the mean outcome after *t* observations (i.e., on trial *t*+1) is simply the average of all outcomes, *O*, that have been observed so far (from trial 1 through trial *t*):

$E_{t+1}={{(O}_{1}+O_{2}+\ldots+O}_{t})/t$ $=\frac{1}{t} \sum_{i=1}^{t} O_{i}$ [S1]

This ‘sample-average’ method can be achieved using a reinforcement-learning algorithm in which the learning rate, $\alpha$, is reduced over trials according to $\alpha_{t}$ = $\frac{1}{t}$, as illustrated by the dotted line in Fig 2B in the main text. This can be seen by rewriting equation S1 as follows:

$E_{t+1}$= $\frac{1}{t} \sum_{i=1}^{t} O_{i}$ = $\frac{1}{t} (O_{t}+\sum_{i=1}^{t-1} O_{i})$ = $\frac{1}{t} (O_{t}+\left( t-1 \right)E_{t})$ = $\frac{1}{t} (O_{t}+tE_{t}-E_{t})$

= $\frac{O_{t}}{t}+ E_{t}-\frac{E_{t}}{t}$ = $E_{t}+\frac{1}{t}(O_{t}-E_{t})$ [S2]

This corresponds to the delta-rule learning algorithm from equation 2 in the main text, with $\frac{1}{t}$ is trial-specific $\alpha$, and $O_{t}-E_{t}$ is prediction error $\delta_{t}$.

Note that this optimal learning-rate regime is approximated by the Kalman filter when (i) the initial prior variance, relative to the noise variance, is set at a very high value (such that the initial learning rate approaches 1) and (ii) the drift variance, relative to the noise variance, is 0 (such that the asymptotic learning rate approaches 0).
