# Supplementary material for: Uncertainty-driven regulation of learning and exploration in adolescents: A computational account
Source: PLoS Comput Biol. 2020 Sep 30;16(9):e1008276. doi: 10.1371/journal.pcbi.1008276 (PMC7549782; doi:10.1371/journal.pcbi.1008276)
Supplement: S3 Text — (DOCX) [file pcbi.1008276.s003.docx]

**Supplementary Text 3: Model recovery analysis**

**Procedure**

We simulated data on each task with each of our models. Each simulated dataset consisted of a group of 25 synthetic participants. The number of blocks and trials per synthetic participant were the same as in the real datasets. For the estimation task we only simulated data from the low-noise condition of the task (standard deviation of outcome-generating distribution = 4)

For each simulation, the hyperparameters governing the model’s group-level distributions—from which the individual-level parameters were drawn—were sampled randomly from uniform distributions. We matched the range of the uniform distributions to the range of values obtained from our fits to the real data, using the minimum and maximum values of the hyperparameters’ posterior medians for the two age groups and (for fits to the estimation data) noise conditions (Supplementary Table 2).

To examine to what extent the data-generating models could be recovered, we applied all models to each simulated dataset and determined the best fitting model for each dataset, using the same model-fitting and comparison procedure as used for the real data. We repeated this procedure 50 times for the estimation data, and 23-29 times for the choice data (the number of repetitions per data-generating model is indicated in parentheses in Supplementary Fig 3B). We used less repetitions for the choice-task simulations for practical reasons: the larger number of models and longer-lasting fitting procedure for this task.

**Results**

We summarize the results in confusion and inversion matrices [1]. Confusion matrices represent the probability that data simulated with a given model is best fit by each of the models, i.e., *p*(fit model | simulated model). For the estimation data, the probability that a dataset simulated with a given model was best fit by that same model, as opposed to one of the other three models, ranged from 0.94 to 1 (Supplementary Fig 3A, left panel). Thus, our procedure could distinguish the learning processes captured by the four different models applied to the estimation data with high accuracy. For the choice data, we found one case in which model-recovery failed: 63% of the datasets generated by the reinforcement learning/Pearce-Hall hybrid model + constant softmax was best fit by the standard RL model + constant softmax. Thus, when the degree of exploration was constant, our method did not identify the adjustment of learning rate according to the Pearce-Hall mechanism, but instead detected a constant learning rate. This is likely due to the low values of decay parameter $\bar{\eta}$ for the reinforcement learning/Pearce-Hall hybrid model + constant softmax (Supplementary Table 2). Such low values of $\bar{\eta}$ produce small decreases in learning rate over time, which could not be dissociated from a constant learning rate. In addition, data generated with the asymmetric RL model + dynamic softmax was best fit by that same model with a probability of .63, and by the standard RL model + dynamic softmax with a probability of .33, indicating moderate identifiability of this model. This may reflect that the values of $\alpha_{+}$ and $\alpha_{-}$ did not differ enough to dissociate asymmetric from symmetric expectation updating. For the other models, the simulated choice data was best fit by the data-generating model, as opposed to one of the other seven models, with probabilities ranging from .75 to .97 (Supplementary Fig 3B, left panel).

To more directly address the question of how to interpret our model-selection results—i.e., how confident can we be that our best-fitting models indeed generated our participants’ data, for which the true underlying model was unknown—we also plotted the inversion matrices (right panels of Supplementary Fig 3). These matrices represent the probability that data that is best fit by a given model is generated by each of the models— *p*(simulated model | fit model)—and can be computed from the confusion matrices using Bayes rule, assuming a uniform prior on models [1]. For the estimation data, the probability that a dataset that was best fit by the Kalman filter (our best-fitting model in both age groups) was indeed generated by that model was .96, validating our model-selection results for the estimation task. For the choice data, the probability that datasets which were best fit by the asymmetric reinforcement learning model + dynamic softmax and the reinforcement learning/Pearce-Hall hybrid model + dynamic softmax (our best-fitting models for the adolescents and adults, respectively) were generated by these same models was .89 and .71, respectively. Thus, we can be rather confident about the model-selection results for the adolescents’ choice data, but should interpret the best-fitting model for the adults’ choice data with some caution. For datasets best fit by the latter model, there was a probability of .19 that they were generated by the standard reinforcement learning model + dynamic softmax. Therefore, we cannot say definitively whether, in the choice task, the adults decreased their learning rate over time according to a Pearce-Hall algorithm or used a constant learning rate. Importantly, the probability that datasets best fit by a dynamic softmax function were indeed generated by a dynamic, instead of a constant, softmax function (regardless of the learning model) ranged from .9 to .97, validating our conclusion that participants in both age groups decreased their degree of exploration over time.
