# Supplementary material for: Uncertainty-driven regulation of learning and exploration in adolescents: A computational account
Source: PLoS Comput Biol. 2020 Sep 30;16(9):e1008276. doi: 10.1371/journal.pcbi.1008276 (PMC7549782; doi:10.1371/journal.pcbi.1008276)
Supplement: S4 Text — (DOCX) [file pcbi.1008276.s004.docx]

**Supplementary Text 4: Parameter recovery analysis**

**Procedure**

We conducted parameter-recovery analyses for the best-fitting models for each task. For the estimation task, we compared the simulated vs. recovered Kalman filter parameters obtained from the model-recovery procedure described above. To further validate the difference in estimated $\bar{\sigma_{\eta}^{2}}$ between the two age groups, we also simulated 100 additional datasets with the Kalman filter, using the posterior medians of $\bar{\sigma_{\eta}^{2}}$ and $\bar{s_{1}^{2}}$ from our fits to the adolescent and adult data (for the low noise condition), for 50 simulations each, and fitted the Kalman filter model to each of these simulated datasets. We then computed 50 difference distributions, one for of each pair of recovered $\bar{\sigma_{\eta}^{2}}$ posteriors, and examined the proportion of pairs for which more than 95% of the difference distribution lay below 0 (i.e., the probability that a group difference was detected in the recovered data).

For the choice task, the range of simulated group-level mean parameters used in the model-recovery analyses was too narrow to examine correlations between simulated and recovered values. Therefore, we simulated 50 additional datasets with the winning model for each age group, using a wider range of parameter values. Specially, group-level mean parameters were randomly sampled from the following uniform distributions: $\bar{\alpha_{+}}$ and $\bar{\alpha_{-}}$~U(.10, .90), $\bar{\alpha_{1}}$~U(.20, .95), $\bar{\eta}$~U(.05, .95),$\bar{\kappa}$~U(.20, .95),$\bar{\theta}$~U(.02, .30),$\bar{c}$~U(.50, .95). Note that we did not simulate values for $\bar{c}$ below .50 because these would produce a decreasing inverse temperature (increase in exploration), which does not correspond to our data or to normative behaviour. Group-level precision parameters were set to the center of the corresponding uniform distributions reported in Supplementary Table 2, in all simulations.

**Results**

The parameter-recovery results of the estimation task revealed a positive correlation between simulated and recovered values of $\bar{\sigma_{\eta}^{2}}$ (the drift variance parameter of the Kalman filter) within the range of the two age groups’ estimated values (*r* = .45, p = .001). However, the recovered values of $\bar{\sigma_{\eta}^{2}}$ were lower than the simulated values, and this bias was larger for higher simulated values (Supplementary Fig 4A, left plot). This suggests that relative, but not absolute, values of $\bar{\sigma_{\eta}^{2}}$ can be interpreted. Simulated and recovered values of $\bar{s_{1}^{2}}$ (the initial prior variance of the Kalman filter) were uncorrelated (*r* = .05, *p* = .72; Supplementary Fig 4A, right plot). This can be explained by the fact that different values of $s_{1}^{2}$ within our high range of simulated values (in the order of hundreds) lead to almost identical behavior. Specifically, all values of $s_{1}^{2}$ > 100 lead to initial learning rates that approach 1 (Equation 3.5 in the main text, note that $\sigma_{\varepsilon}^{2}$ was set to 1), and to an almost identical adjustment of the prior variance—and hence learning rate—on subsequent trials (Equation 3.2 in the main text). Recovered values of $\bar{\sigma_{\eta}^{2}}$ and $\bar{s_{1}^{2}}$ were uncorrelated (*r* = .02, *p* = 88), indicating that there was no trade-off between the two Kalman-filter parameters.

Our follow-up analysis that specifically addressed the difference in $\bar{\sigma_{\eta}^{2}}$ between the two age groups showed that the posterior median of $\bar{\sigma_{\eta}^{2}}$ from fits to the adults’ estimation data (.00009) was well recovered, while the estimated value of $\bar{\sigma_{\eta}^{2}}$ from fits to the adolescents’ data (.02) was strongly underestimated (Supplementary Fig 4B). Recovered $\bar{\sigma_{\eta}^{2}}$ was numerically higher for data simulated with the estimated values from the adolescent than the adult group in all simulations, but the recovered group difference was smaller than the simulated difference. For 70% of the simulations, more than 95% of the recovered difference distribution ($\bar{\sigma_{\eta}^{2}}$ adults – $\bar{\sigma_{\eta}^{2}}$ adolescents) lay below 0. These findings suggest that the ‘real’ value of $\bar{\sigma_{\eta}^{2}}$ for the adolescent group is likely to be higher—and the age-related difference in $\bar{\sigma_{\eta}^{2}}$ larger—than it seemed based on the model fits to the empirical data.

Regarding the choice-task models, simulated and recovered group-level mean parameters from the asymmetric reinforcement learning model + dynamic softmax—the winning model for the adolescents—were strongly correlated (Supplementary Fig 5A), and there was no evidence for bias, or for tradeoffs between parameters (all correlations between recovered parameters < .25, *p*’s > .08). For the reinforcement learning/Pearce-Hall hybrid model + dynamic softmax—the winning model for the adults—simulated and recovered hyperparameters were also correlated. However, $\bar{\eta}$, $\bar{\kappa}$ and $\bar{c}$ tended to be underestimated, whereas $\bar{\theta}$ tended to be overestimated for this model (Supplementary Fig 5B). In addition, $\bar{\kappa}$ (constant component of the learning rate) and $\bar{\theta}$ (inverse temperature halfway a task block) were negatively correlated (*r* = -.43, *p* = .002), reflecting the typical tradeoff between learning rate and inverse temperature [2].
