# Supplementary material for: Uncertainty-driven regulation of learning and exploration in adolescents: A computational account
Source: PLoS Comput Biol. 2020 Sep 30;16(9):e1008276. doi: 10.1371/journal.pcbi.1008276 (PMC7549782; doi:10.1371/journal.pcbi.1008276)
Supplement: S1 Fig — (DOCX) [file pcbi.1008276.s005.docx]

**Supplementary Figure 1.** Control analyses on the dynamic-softmax parameters. We repeated the group comparisons of $\bar{\theta}$, $\bar{c}$, and inverse temperature reported in the main text, this time using the parameter estimates derived from the same model in both age groups: the reinforcement learning/Pearce-Hall hybrid model + dynamic softmax. **A**. Posterior distributions for the group-level central tendencies of the dynamic-softmax parameters per age group (left panels) and the corresponding difference distributions (right panels*)* for fits to the choice data (corresponding to Fig. 5D in the main text). **B**. Model’s predicted inverse temperature per trial (corresponding to Fig 5E, right panel, in the main text).

**
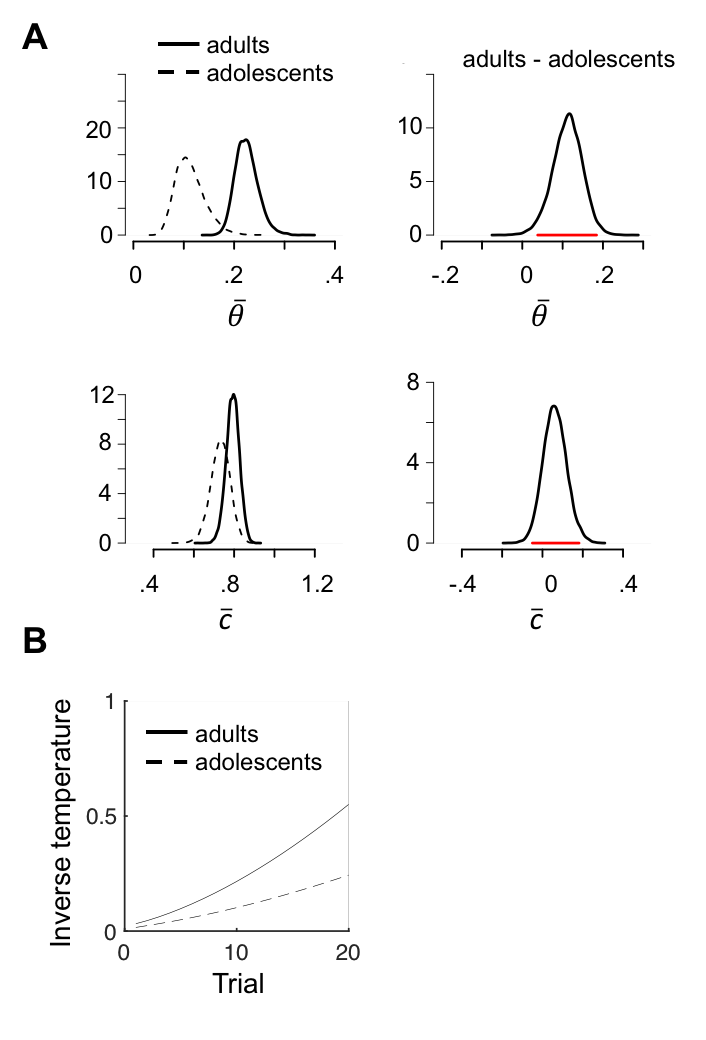
**
