# Supplementary material for: Uncertainty-driven regulation of learning and exploration in adolescents: A computational account
Source: PLoS Comput Biol. 2020 Sep 30;16(9):e1008276. doi: 10.1371/journal.pcbi.1008276 (PMC7549782; doi:10.1371/journal.pcbi.1008276)
Supplement: S2 Fig — (DOCX) [file pcbi.1008276.s006.docx]

**Supplementary Fig 2**. Control analyses on the relationships between age group and model parameters $\sigma_{\eta}^{2}$, $\theta$ and *c*. We repeated the mediation analyses reported in the main text (Fig 6), this time using $\theta$ and *c* estimates derived from the same choice-task model in both age groups **A**. Mediation models and results. **B**. Across-subject relationships between $\sigma_{\eta}^{2}$ and $\theta$, and between $\sigma_{\eta}^{2}$ and *c* (all converted to ranks), controlled for age group (path *b* of the mediation models). The partial correlations are *r* = -.34, *p* = .01 *r* = -.47, *p* < .001, respectively.

**
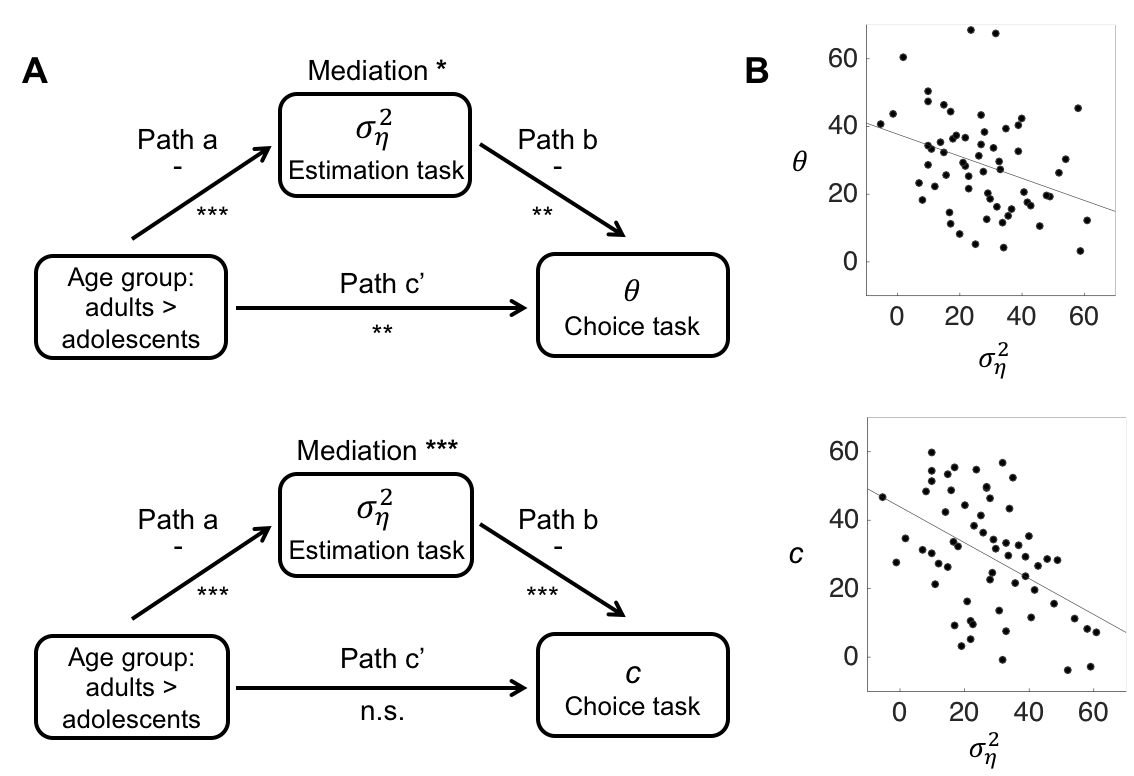
**
