# Supplementary material for: Uncertainty-driven regulation of learning and exploration in adolescents: A computational account
Source: PLoS Comput Biol. 2020 Sep 30;16(9):e1008276. doi: 10.1371/journal.pcbi.1008276 (PMC7549782; doi:10.1371/journal.pcbi.1008276)

**Supplementary Fig 3.** Model-recovery results for the estimation (**A**) and choice (**B**) task. For the estimation data, we simulated 50 datasets from each model. For the choice task, the number of simulated datasets is indicated in parentheses. Models 1-4 are the reinforcement-learning, asymmetric reinforcement-learning, Kalman-filter, and reinforcement learning/Pearce-Hall hybrid models, respectively. Version a and b of each learning model (in Fig B) are combined with the constant and dynamic softmax function, respectively.


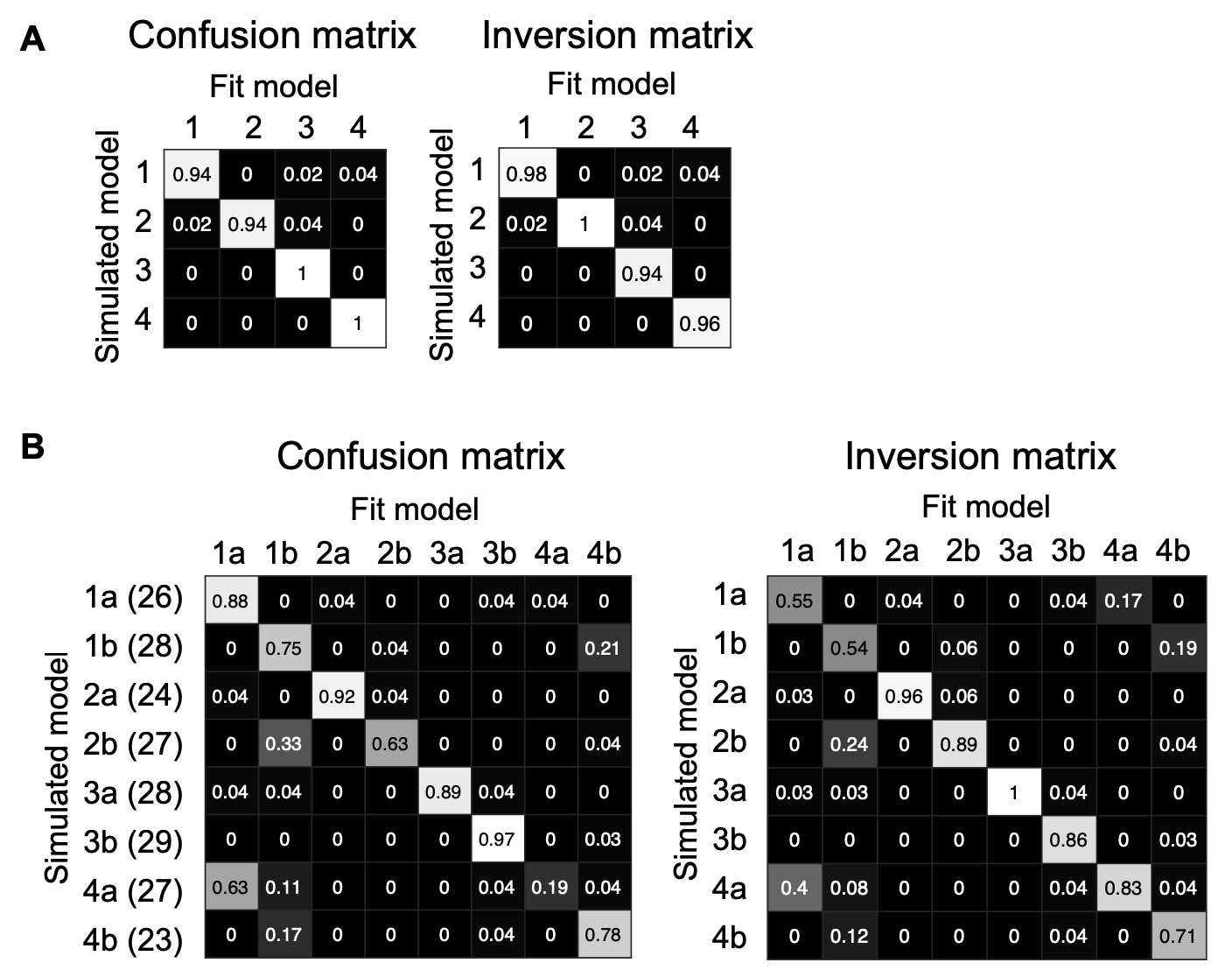

Supplement: S3 Fig — (DOCX) [file pcbi.1008276.s007.docx]
