# Supplementary material for: Uncertainty-driven regulation of learning and exploration in adolescents: A computational account
Source: PLoS Comput Biol. 2020 Sep 30;16(9):e1008276. doi: 10.1371/journal.pcbi.1008276 (PMC7549782; doi:10.1371/journal.pcbi.1008276)
Supplement: S4 Fig — (DOCX) [file pcbi.1008276.s008.docx]

**Supplementary Fig 4**. Parameter-recovery results for the Kalman filter in the estimation task. **A**. Simulated vs. recovered $\bar{\sigma_{\eta}^{2}}$ and $\bar{s_{1}^{2}}$. Simulated values for both hyperparameters were randomly drawn from a uniform distribution which range matched the range of values from our fits to the real data. Black lines are regression lines, and red lines are lines of equality (simulated = recovered). **B**. Recovered $\bar{\sigma_{\eta}^{2}}$ when simulated hyperparameters were set to the estimated values for the adolescent (green) and adult (purple) group, 50 times each.

**
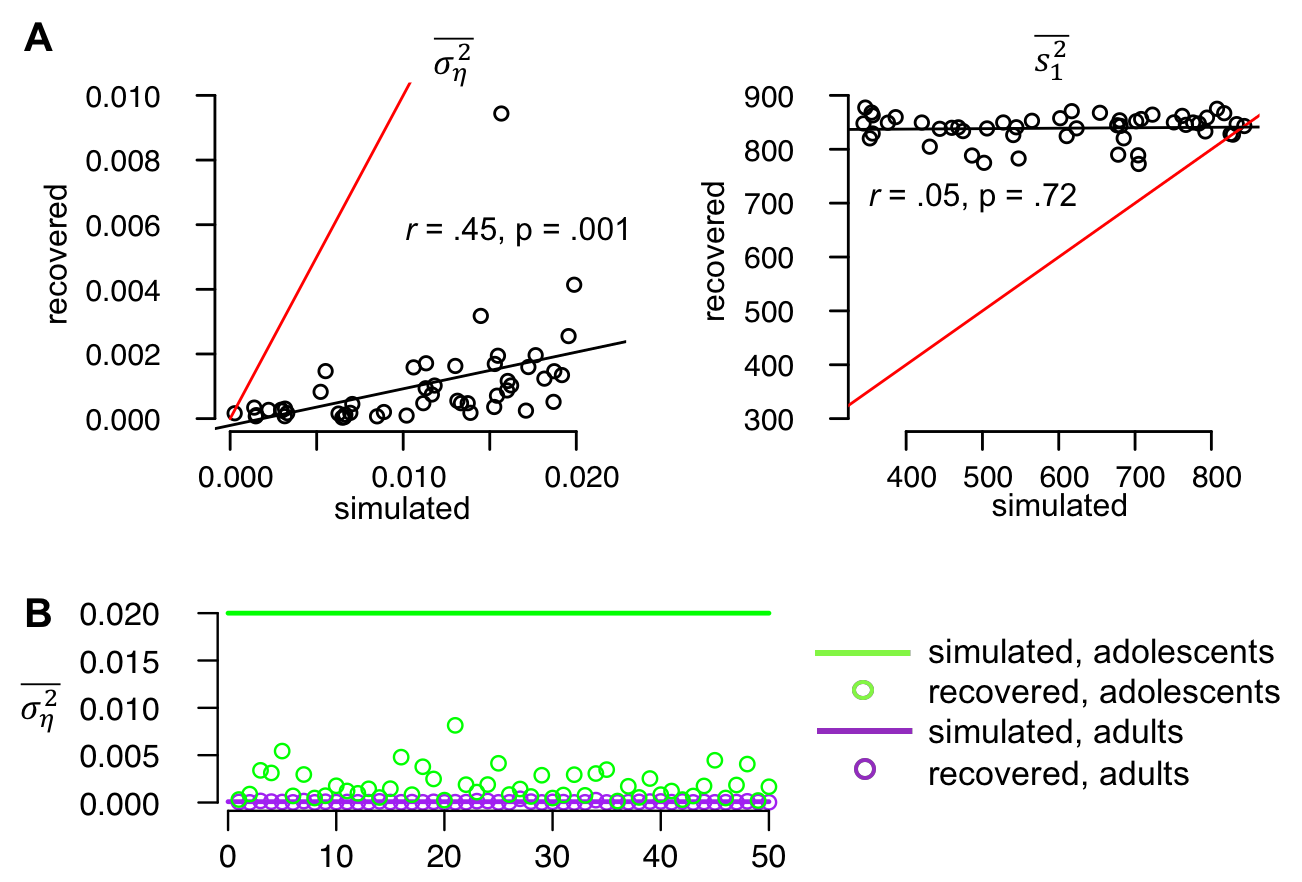
**
