# Supplementary material for: Uncertainty-driven regulation of learning and exploration in adolescents: A computational account
Source: PLoS Comput Biol. 2020 Sep 30;16(9):e1008276. doi: 10.1371/journal.pcbi.1008276 (PMC7549782; doi:10.1371/journal.pcbi.1008276)
Supplement: S5 Fig — (DOCX) [file pcbi.1008276.s009.docx]

**Supplementary Fig 5**. Parameter-recovery results for the best-fitting models in the choice task. **A**. Simulated vs. recovered group-level mean parameters of the asymmetric reinforcement-learning model + dynamic softmax. **B**. Simulated vs. recovered group-level mean parameters of the reinforcement learning/Pearce-Hall hybrid model + dynamic softmax. Red lines are lines of equality.**
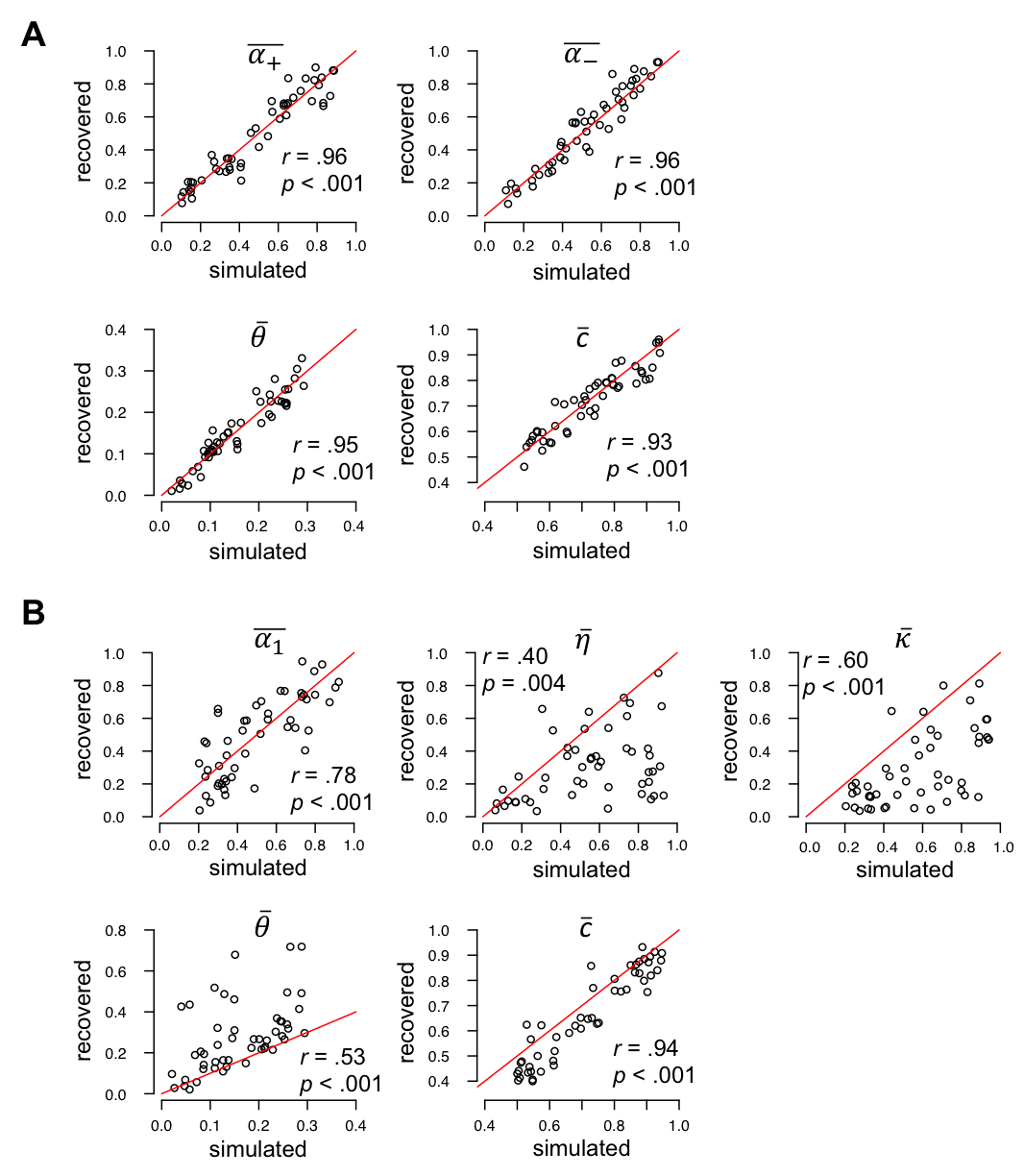
**
