# Supplementary material for: Uncertainty-driven regulation of learning and exploration in adolescents: A computational account
Source: PLoS Comput Biol. 2020 Sep 30;16(9):e1008276. doi: 10.1371/journal.pcbi.1008276 (PMC7549782; doi:10.1371/journal.pcbi.1008276)
Supplement: S1 Table — (DOCX) [file pcbi.1008276.s010.docx]

**Supplementary Table 1.** The average probability of participants’ choices (standard deviations in parentheses) under each model.

|  | adults | adolescents |
| --- | --- | --- |
| Model 1A: RL/constant $\beta$ | 0.76 (0.06) | 0.64 (0.12) |
| Model 1B: RL/dynamic $\beta$ | 0.78 (0.07) | 0.65 (0.13) |
| Model 2A: RL2/constant $\beta$ | 0.76 (0.06) | 0.64 (0.12) |
| Model 2B: RL2/dynamic $\beta$ | 0.78 (0.06) | 0.65 (0.13) |
| Model 3A: KF/constant $\beta$ | 0.76 (0.06) | 0.64 (0.12) |
| Model 3B: KF/dynamic $\beta$ | 0.78 (0.07) | 0.65 (0.13) |
| Model 4A: PH/constant $\beta$ | 0.56 (0.09) | 0.54 (0.05) |
| Model 4B: PH/dynamic $\beta$ | 0.78 (0.07) | 0.65 (0.13) |

Notes: we computed the probability of each choice, separately for each participant, using the posterior medians of the individual-level parameters and the outcomes observed up to that choice. We then averaged probabilities across trials and participants. Standard deviations were based on each participant’s trial-average probability. $\beta$ = inverse temperature.
