# Supplementary material for: Uncertainty-driven regulation of learning and exploration in adolescents: A computational account
Source: PLoS Comput Biol. 2020 Sep 30;16(9):e1008276. doi: 10.1371/journal.pcbi.1008276 (PMC7549782; doi:10.1371/journal.pcbi.1008276)
Supplement: S2 Table — (DOCX) [file pcbi.1008276.s011.docx]

**Supplementary Table 2**. Medians (and 95% highest density intervals) of the posterior distributions shown in Fig 5A, C and D in the main text.

|  | Estimation task | | | |
| --- | --- | --- | --- | --- |
|  | Low noise | | High noise | |
|  | Adults | Adolescents | Adults | Adolescents |
| $\bar{\sigma_{\eta}^{2}}$ | .00009 (.000003 - .0004) | .02 (.0007 - .08) | .00003 (.000001 - .0001) | .001 (.00001 - .006) |
| $\bar{s_{1}^{2}}$ | 819 (469 - 1,000) | 844 (512 - 1,000) | 342 (68 – 831) | 778 (382 - 1,000) |
|  | Choice task | | | |
|  | Adults | | Adolescents | |
| $\bar{\alpha_{1}}$ | .91 (.71 – 1.0) | |  | |
| $\bar{\eta}$ | .12 (.015 - .23) | |  | |
| $\bar{\kappa}$ | .73 (.58 - .96) | |  | |
| $\bar{\alpha_{+}}$ |  | | .52 (.32 - .71) | |
| $\bar{\alpha_{-}}$ |  | | .49 (.34 - .65) | |
| $\bar{\theta}$ | .22 (.18 - .27) | | .14 (.08 - .21) | |
| $\bar{c}$ | .79 (.73 - .86) | | .74 (.64 - .85) | |
