# Supplementary material for: Uncertainty-driven regulation of learning and exploration in adolescents: A computational account
Source: PLoS Comput Biol. 2020 Sep 30;16(9):e1008276. doi: 10.1371/journal.pcbi.1008276 (PMC7549782; doi:10.1371/journal.pcbi.1008276)
Supplement: S3 Table — (DOCX) [file pcbi.1008276.s012.docx]

**Supplementary Table 3.** Prior distributions for the simulation hyperparameters used in the model-recovery analysis.

| Model | Prior distributions |
| --- | --- |
| RL | $\alpha^{mean}$~U(.56, .85), $\alpha^{prec}$~ U(6, 14) |
| RL2 | $\alpha_{+}^{mean}$~U(.56, .83), $\alpha_{+}^{prec}$~ U(4, 11), $\alpha_{-}^{mean}$~U(.54, .83), $\alpha_{-}^{prec}$~ U(6, 21) |
| KF | $\sigma_{\eta}^{2 scale}$ ~ U(.00003, .02), $s_{s,1}^{2 scale}$~ U(342, 844) |
| PH | $\alpha_{1}^{mean}$~U(.97, .99),$\alpha_{1}^{prec}$~U(6, 29), $\eta^{mean}$~U(.25, .39), $\eta^{prec}$~U(3, 4), $\kappa^{mean}$~U(.96, .99), $\kappa^{prec}$~U(4, 33) |
| RL/con expl | $\alpha^{mean}$~U(.42, .43), $\alpha^{prec}$~ U(5, 6), $\beta^{mean}$~U(.11, .20), $\beta^{prec}$~ U(8, 6) |
| RL/dyn expl | $\alpha^{mean}$~U(.55, .59), $\alpha^{prec}$~ U(6, 8), $\theta^{mean}$~U(.12, .22), $\theta^{prec}$~ U(4, 17),  $c^{mean}$~U(.72, .78), $c^{prec}$~ U(5.5, 5.8) |
| RL2/con expl | $\alpha_{+}^{mean}$~U(.25, .26), $\alpha_{+}^{prec}$~ U(7,13), $\alpha_{-}^{mean}$~U(.46, .51), $\alpha_{-}^{prec}$~ U(2.5, 2.8),  $\beta^{mean}$~U(.18, .22), $\beta^{prec}$~ U(5, 45) |
| RL2/dyn expl | $\alpha_{+}^{mean}$~U(.52, .61), $\alpha_{+}^{prec}$~ U(3, 5), $\alpha_{-}^{mean}$~U(.49, .60), $\alpha_{-}^{prec}$~ U(2, 3),  $\theta^{mean}$~U(.14, .22), $\theta^{prec}$~ U(3, 17), $c^{mean}$~U(.74, .79), $c^{prec}$~ U(5, 6) |
| KF/con expl | $\sigma_{\eta}^{2 scale}$ ~ U(.04, .11), $s_{s,1}^{2 scale}$~ U(.05, .14),$\beta^{mean}$~U(.11, .21), $\beta^{prec}$~ U(6, 52) |
| KF/dyn expl | $\sigma_{\eta}^{2 scale}$ ~ U(.06, .28), $s_{s,1}^{2 scale}$~ U(16, 231), $\theta^{mean}$~U(.10, .21), $\theta^{prec}$~ U(5, 16), $c^{mean}$~U(.75, .80), $c^{prec}$~ U(4.8, 5.2) |
| PH/con expl | $\alpha_{1}^{mean}$~U(.68, .71),$\alpha_{1}^{prec}$~U(4, 11), $\eta^{mean}$~U(.04, .05), $\eta^{prec}$~U(3, 7), $\kappa^{mean}$~U(.61, .62), $\kappa^{prec}$~U(4, 8),$\beta^{mean}$~U(.11, .20), $\beta^{prec}$~ U(7, 70) |
| PH/dyn expl | $\alpha_{1}^{mean}$~U(.86, .91),$\alpha_{1}^{prec}$~U(10, 16), $\eta^{mean}$~U(.07, .12), $\eta^{prec}$~U(2, 9), $\kappa^{mean}$~U(.73, .84), $\kappa^{prec}$~U(5, 9),$\theta^{mean}$~U(.11, .22), $\theta^{prec}$~ U(5, 15), $c^{mean}$~U(.73, .79), $c^{prec}$~ U(5, 6) |

Notes: The first four model were used to simulate estimation data and the last four models to simulate choice data. RL, RL2, KF, and PH are the reinforcement-learning, asymmetric reinforcement-learning, Kalman-filter, and reinforcement learning/Pearce-Hall hybrid model, respectively, dyn = dynamic, con = constant, expl = exploration, prec = precision (variance^-1^)
