# Supplementary material for: Uncertainty-driven regulation of learning and exploration in adolescents: A computational account
Source: PLoS Comput Biol. 2020 Sep 30;16(9):e1008276. doi: 10.1371/journal.pcbi.1008276 (PMC7549782; doi:10.1371/journal.pcbi.1008276)
Supplement: Attachment — Submitted filename: ReplyToReviewers_Jepma_etal.docx [file pcbi.1008276.s013.docx]

**Summary**

We are grateful to the reviewers for their positive comments and insightful criticisms of our manuscript. To respond to the issues raised, we have extended our model-based analyses in several ways. Most importantly, we (i) extended our model space with two additional models to examine potential alternative explanations for the observed age-related differences in learning rate and exploration, and (ii) conducted model-recovery and parameter-recovery analyses. We have also made a number of other clarifications and additions to accommodate the reviewers’ suggestions.

Below, the reviewers’ comments are typed in italics, our replies in normal (black) font, and the changes/additions we made in the manuscript in blue.

**Reviewer #1**

*I think the paper has the potential to deliver an important message to the cognitive developmental community, however, I believe that several issues have to be addressed in order to support their claims.

1/ the first point concerns the model space. The authors limit model comparison to a standard Rescorla-Wagner model and the Kalman-filter model. Learning in this kind of situations have been studied extensively and a lot of (psychologically sound) models have been proposed and validated. They should be included in the model space. These models include: An asymmetric reinforcement learning model (see Lefebvre et al.2017 and Sharot’s work) for we which we have good reasons to think that there will be differences between adults and adolescents (see Van Den Bos, Cerebral Cortex, 2013). My prediction is that the noise variance parameter will significantly correlate with the difference in learning rates in this model. A Pearce-Hall model (which is a simpler, non Baysian, instantiation of the idea that the learning rate is dynamically modulated).*

Reply:

We extended our model space with the two models proposed by the Reviewer (asymmetric RL model and Pearce-Hall model). For fits to the choice data, we combined each of these learning models with a constant and a dynamic softmax function. The additional models are summarized on p. 15 and 16-17 of the Results section:

*Model 2: asymmetric reinforcement-learning model*

This model is identical to Model 1, except that it uses two separate learning rates to control expectation updating following positive and negative prediction errors: $\alpha_{+}$and $\alpha_{-}$, respectively. Thus, this model has two free parameters. This model may explain age-related differences in choice behaviour in terms of differential sensitivity to better- and worse-than-expected outcomes [3, 9, 10]. Note that a learning asymmetry is less likely in the estimation task, as positive and negative prediction errors in this task do not reflect better- and worse-than-expected outcomes, respectively. However, for completeness, we applied all models to both tasks.

*Model 4: reinforcement learning/Pearce-Hall hybrid model*

This model also has a dynamic learning rate, but the learning rate on each trial is defined in a non-Bayesian way, by scaling it by a dynamic ‘associability’ term which reflects the unexpectedness of previous outcomes [28]. Specifically, the associability on a given trial, $\alpha_{t}$, is a weighted average of the associability and the absolute prediction error on the previous trial. Decay parameter $\eta$ determines the relative weights of these two terms. This model can be seen as a hybrid between the standard reinforcement learning model and the Pearce-Hall associability model [29-31]. It has three free parameters: the initial associability $\alpha_{1}$, decay parameter$\eta$, and the constant component of the learning-rate $\kappa$ (the learning rate on trial *t* is the product of$\kappa$ and $\alpha_{t}$).

We provide their equations on p. 39 and 41 of the Methods section:

*Asymmetric reinforcement-learning model*

This model is identical to the standard reinforcement learning model, with the following exception: it uses two separate learning rates ($\alpha_{+}$ and $\alpha_{-}$) to update expectations following positive and negative prediction errors, hence Equation 1.2 is replaced by:

$E_{s,t+1}=\left\{ \begin{aligned} E_{s,t}+ \alpha_{+}\delta_{s,t} \mathrm{if}\delta_{s,t}>0 \\ E_{s,t}+ \alpha_{-}\delta_{s,t}\mathrm{if}\delta_{s,t}<0 \end{aligned} \right.$	[2.1]

*Reinforcement learning/Pearce-Hall hybrid model*

This model scales the learning rate by a dynamic ‘associability’ term which originates from the Pearce-Hall model [28]. The associability on trial *t*, $\alpha_{t}$, is a weighted average of the absolute prediction error and associability on the previous trial:

$\alpha_{s,t}=\eta\left| \delta_{s,t-1} \right| +\left( 1-\eta\right)\alpha_{s,t-1}$	[4.1]

Decay parameter $\eta$ determines the relative weights of these two terms. The associability term is incorporated in the expectation updating algorithm from the standard reinforcement learning model [29-31]:

$E_{s,t+1}=E_{s,t}+\kappa\alpha_{s,t}\delta_{s,t}$	[4.2]

The initial associability (on trial 1; $\alpha_{1}$) is estimated as a free parameter. Thus, this model has three free parameters: $\alpha_{1}$, $\eta$, and $\kappa$.

For fits to the estimation data, we found that the Kalman filter was still the winning model for both age groups. However, the model-selection results for the choice task changed: the RL/Pearce-Hall hybrid model + dynamic softmax best explained the adults’ choices, whereas the asymmetric RL model + dynamic softmax best explained the adolescents’ choices. The new model-comparison results are reported on p. 19 of the Results and in revised Table 1:

For fits to the choice data, model 4B (the reinforcement learning/Pearce-Hall hybrid model + dynamic softmax) performed best for the adults, while model 2B (asymmetric reinforcement-learning model + dynamic softmax) performed best for the adolescents. This suggests that when making repeated choices between two options—as compared to estimating the average value of one stimulus—adults use a simpler, non-Bayesian, mechanism to adjust their learning rate over time, while adolescents do not adjust their learning rate over time. Importantly, all learning models performed better when combined with a dynamic than a constant softmax function, for both age groups, suggesting that both the adults and adolescents did adjust their degree of exploration over time.

We also edited the abstract to accommodate this change of results, and added the following sentence (p. 2):

In contrast to the estimation task, we found no evidence for a Bayesian expectation-updating process in the choice task, suggesting that estimation and choice tasks engage different learning processes.

*Another important model (even if harder to fit) is the noisy-RL model recently proposed by Findling et al. (nature neuroscience), as this noisy update could explain softmax modulations.*

Reply:

We agree that the recently proposed noisy-RL model is another relevant model. However, because of its more complex fitting procedure (for which Python and C++, but no R/JAGS, code is available), we feel that adding this model is beyond the scope of the current manuscript. However, we discuss the potential contribution of learning noise to our findings in the revised Discussion (p. 31-32):

Finally, a recent study in adults showed that, in a restless two-armed bandit task, a substantial proportion of choices for the lower-valued option can be explained by internal computational noise in the expectation-updating process—resulting in random fluctuations in expected outcome values—rather than by exploratory choice behaviour [56]. An interesting idea that follows—and remains to be tested—is that not only learning rate and exploration, but also ‘learning noise’, increase as a function of environmental volatility. If this is the case, adolescents’ increased representation of volatility could have resulted in a noisier learning process in this age group. Whether, and to what extent, age-related differences in learning noise may underlie the age-related effects on learning rate and exploration found in the present study needs to be examined in future work, for example using the recently developed ‘noisy RL model’ [56].

*2/ the second point is that the model-comparison results (concerning the old and the new models) should be backed-up by a model recovery analysis, where the authors show that, in simulated datasets, they are capable to retrieve the correct, generative, model. The procedure should be extended to the parameters of the best fitting model to ensure that the conclusions based on parameters comparison are justified.*

Reply:

We conducted model-recovery analyses and report the results in confusion and inversion matrices (Supplementary Figure 3). Results are summarized on p. 26 of the main text, and the procedure and results are described in detail in Supplementary text 3. In addition, we conducted parameter-recovery analyses for the best-fitting models. Their results are summarized on p. 27 of the main text and described in more detail in Supplementary Text 4 and Supplementary Figures 4-5. These are the summaries of both sets of analyses in the main text (p. 26-27):

*Validation of modeling results*

To validate our model-selection and parameter-estimation results, we performed model-recovery and parameter-recovery analyses, respectively. We describe the main outcomes here; procedures and full results are provided in the Supplementary Material (Supplementary Text 3-4; Supplementary Figures 3-5).

*Model recovery.* Model-recovery results indicate that our procedure could distinguish the learning and choice processes implemented in our different models with rather high accuracy, with one exception: a high proportion of choice datasets generated by the reinforcement learning/Pearce-Hall hybrid model + constant softmax was best fit by the standard reinforcement model + constant softmax (discussed in Supplementary Text 3; Supplementary Figure 3). However, these models were never the best-fitting models in our analysis of the empirical data. Regarding our best-fitting models, the results indicate that (i) we can be 94% confident about the best-fitting model for the estimation task (Kalman filter, in both age groups); (ii) we can be 89% confident about the best-fitting model for the choice task in the adolescents (asymmetric reinforcement learning model + dynamic softmax); (iii) we can be 71% confident about the best-fitting model for the choice task in the adults (reinforcement learning/Pearce-Hall hybrid model + dynamic softmax); and (iv) we can be 90-97% confident about the best fitting softmax function in both age groups (dynamic softmax).

*Parameter recovery.* For the estimation-task models, our parameter-recovery results corroborate the presence of an age-related difference in the Kalman filter’s drift-variance parameter $\sigma_{\eta}^{2}$. However, while values of $\bar{\sigma_{\eta}^{2}}$ corresponding to the estimated value for the adult group were accurately recovered, values of $\bar{\sigma_{\eta}^{2}}$ corresponding to the estimated value for the adolescent group tended to be underestimated (Supplementary Figure 4). Thus, the actual value of $\bar{\sigma_{\eta}^{2}}$ for the adolescent group may well have been higher—and hence the age-related difference in $\bar{\sigma_{\eta}^{2}}$ larger—than it seemed based on the parameter estimates from our model fits to the empirical data.

Regarding the choice-task models, all group-level mean parameters of the asymmetric reinforcement learning model + dynamic softmax—the best-fitting model for the adolescents—were well recovered (correlations between simulated and recovered values > .92, *p*’s < .001, for all parameters; Supplementary Figure 5A), with no evidence for bias or tradeoffs between parameters. For the reinforcement learning/Pearce-Hall hybrid model + dynamic softmax—the best fitting model for the adults—simulated and recovered parameters were correlated as well (correlations range from .4 to .94, all *p*’s < .005). However, $\bar{\eta}$, $\bar{\kappa}$ and $\bar{c}$ tended to be underestimated, whereas $\bar{\theta}$ tended to be overestimated for this model (Supplementary Figure 5B). Thus, the estimated values of these hyperparameters should be taken with some caution.

*3/ it is unclear which aspect of the behaviour (especially in the bandit task) IS NOT captured by the standard model. The only model-free results that are displayed are the learning curves (figure 4) and this difference can be perfectly captured by a standard model. Is there more information in the behaviour that can help us discriminate the models?*

Reply:

We agree that it would be good to look for additional, model-free, evidence for dynamic changes in exploration (and age-related differences in this) in the bandit task. We thought about possible ways to do this, and came up with the following: A model-free way to define exploitation vs. exploration in this task is to classify choices for the option with the highest and lowest average observed outcome (observed during the trials preceding the current choice) as exploitative and exploratory, respectively. If people’s tendency to explore is constant over time (standard softmax function), their proportion of exploratory choices should not change over trials, when controlled for the difference in the options’ average observed outcome. In contrast, if people become less exploratory over time (dynamic softmax function), their proportion of exploratory choices should decrease over trials. This additional analysis and its results are added on p. 13-14:

To examine initial evidence for changes in exploration over time (see the next section for model-based analyses), we classified each choice as exploitative or exploratory in the following way. For each trial, we computed the average outcome that had been observed for each option so far (a model-free estimate of expected value), and we classified choices for the option with the higher and lower expected value as, respectively, exploitative and exploratory (only including the trials at which both options had been chosen at least once before). We then tested for main and interaction effects of trial (linear and quadratic) and age group on exploratory choices, as defined above. Importantly, to control for changes in relative expected value over time, we also modeled the absolute difference between the two options’ expected value. As expected, exploration decreased as the difference in expected value increased (*z* = 5.0, *p* < .001). This effect of relative expected value on exploration was larger in the adults than the adolescents (*z* = 4.9, *p* < .001). This analysis also revealed a linear decrease in exploration over trials (*z* = 5.8, *p* < .001). Finally, the adolescents, as compared to the adults, showed more exploration overall (*z* = 4.7, *p* < .001) as well as a smaller linear decrease in exploration over trials (*z* = 4.3, *p* < .001). These findings provide initial evidence that participants—and adults more so than adolescents—reduced their degree of exploration over time, consistent with normative behaviour on this task.

*4/ it would be important to know what is the correlation between the parameters estimated in the estimation and in the learning task. Is there some common ground? Are the two tasks tapping into the same processes? I suspect that they don’t and that different models (and different biases) apply to the two tasks.*

Reply:

Our extended modeling results indeed suggest that participants used different learning processes in the estimation and choice task. Specifically, the Kalman filter best explained both age groups’ estimation data, while the asymmetric RL model (+ dynamic softmax function) and the Pearce-Hall model (+ dynamic softmax function) best explained the adolescents’ and adults’ choice data, respectively (see our reply to the first comment). This suggests that when people make repeated choices between two stimuli, they use simpler (non-Bayesian) expectation-updating processes than when they estimate the mean outcome associated with one stimulus. We discuss this important point in the revised Discussion (p. 30-31):

By directly comparing an estimation and a choice task, our study also provides evidence that estimating the mean value of a stimulus engages a different learning process than making choices between two stimuli. The estimation data of both age groups was best described by a learning model that integrates new outcomes and prior expectations using Bayesian inference (Kalman filter). In contrast, the choice data was best described by simpler, non-Bayesian, learning processes (reinforcement learning/Pearce-Hall hybrid model for the adults, and asymmetric reinforcement learning model for the adolescents). Thus, during the choice task, we found evidence that adults decreased their learning rate over time—although in a different way than in the estimation task—whereas adolescents used constant learning rates. Possible reasons for the use of simpler, non-Bayesian, learning processes in the choice task are that (i) optimal performance in this task requires estimating the *relative* rather than the *exact* value of stimuli and/or (ii) optimal choice performance can be achieved by a simpler updating process when combined with a dynamic adjustment of the exploration-exploitation tradeoff. In addition, the choice task involved learning about two stimuli in parallel (compared to one stimulus in the estimation task), requiring more working-memory resources, which may have resulted in the use of simpler learning processes. Indeed, estimation performance on a more computationally demanding estimation task in a previous study—adult participants learned about two stimuli during each task block—was best explained by a non-Bayesian learning model that resembled our best-fitting model for the adults’ choice data [47].

*5/ it is important to provide the instructions given to the subjects, as it is important to know whether or not the subjects were primed about the frequent changes in contingencies. This would change the interpretation of the results, as it could simply be that adolescents do not follow instructions.*

Reply:

We describe the instructions given to the subjects for each task in the Methods (p. 35, 36). Importantly, participants were informed that outcome contingencies did not change within task blocks, but did vary between task blocks. We now make this clear earlier on, in the beginning of the Results section (p. 8):

Except for the specific means and SDs used, participants were fully informed about the task structure and procedure before starting each task (also see Methods).

Also, the start of each new block was clearly indicated to the participants, which we mention in the revised methods (p. 34):

The start of each new block was clearly indicated, by means of a ‘new-block’ screen, and a different box, with a unique color, was used in each block.

*6/ what is the rational to fix the noise variance parameter? How does its inclusion change the results (I guess it favour the Kalman filter because it reduces its number of free parameters)?*

Reply:

Only the ratio between the 3 variance parameters of the Kalman filter matters; hence one of them has to be fixed (otherwise the model is not identifiable). The model fit does not depend on which of the 3 parameters is fixed.
 *7/ how have the authors verified that the two groups were matched in terms of IQ and socio-economic status?*

Reply:

We matched the two groups in terms of educational level—all participants followed, or had followed, (pre-) university or higher professional education—as mentioned in the Methods (p. 33):

Adults were students, or former students, at universities or colleges of higher professional education. Adolescents attended high schools (pre-university or higher general secondary education).

To make this clear earlier on, we also added this in the beginning of the Results section (p. 7): Participants (35 adults and 25 adolescents; matched with regard to educational level)

**Reviewer #2**

*I found this paper very well written, with clear exposition of the computational terms and models, and a compelling set of results. I was especially impressed by the explanations of the Kalman Filter, which are the clearest I have encountered. The experiments are clean and well chosen, and the results described in detail, with well-thought-out statistical analyses.

My main worry regards the discrepancy between the behavioral results and the conclusions from the modeling: while the behavioral results suggested no age-related differences in uncertainty and how it changed over time, and in the process of updating learning rates with experience, the results of the modeling were considered as suggesting the opposite. That is, based on the modeling results, it was suggested that adolescents assume more variation in the outcome-generating process over time, which drives their high learning rate and suboptimal choice behavior. First, since the models tested had only one means by which to generate a higher learning rate (the Kalman Filter determines learning rate based on estimated volatility and observation noise), and we know empirically that the asymptotic learning rate was higher for adolescents, I am not sure I buy this explanation of the provenance of the higher learning rate. At the very least it should be clarified that this was by design of the model -- another model, that explained higher asymptotic learning rates differently, might have suggest a different cause.*

Reply:

Thanks for this important comment. First, we agree that the idea that adolescents assume more variation in the outcome-generating process than adults seems at odds with the absence of age effects on self-reported certainty. We acknowledge this and suggest possible explanations for this apparent discrepancy in the Discussion (p. 29-30; the first three possible explanations were already in the original submission, we added the last one):

What might explain this apparent discrepancy? First, this may be explained by the interdependence of volatility and noise described above: If adolescents assume more volatility than adults, but also less noise, this could result in increased learning rates (which scales with the ratio between volatility and noise) despite the same ‘overall’ certainty experience (which scales with their sum). Second, the evaluation of the various forms of uncertainty is likely to be an (at least partially) implicit process. Indeed, our model-based analyses revealed that participants (especially the adolescents) assumed variability in outcome contingencies over time even though they were instructed that this was not the case, suggesting a dissociation between participants’ explicit task knowledge and the internal model they used to perform the task. Third, it has been proposed that self-evaluation of performance involves a distinct decision system that “reads out” confidence information from internal states, and that substantial bias can be introduced during this process [38, 39]. Thus, age-related differences in this reporting bias may also explain why adolescents reported to be as certain as adults while their learning rates suggested otherwise. Finally, that the adolescents reported to be equally certain as the adults, even though their behaviour suggested that they judged the environment to be more volatile, may indicate that the adolescents were actually more confident about their performance than the adults. This last possibility is broadly consistent with some previous developmental studies on performance monitoring [45, 46].

Furthermore, in the revised manuscript we explicitly look for alternative explanations for the observed age differences in learning rate and choice accuracy, by applying two additional models: an RL/Pearce-Hall hybrid model (which can explain changes in learning rate over time in a non-Bayesian way) and an asymmetric RL model (which may explain differences in choice behavior by differential sensitivity to positive vs. negative prediction errors). The additional models are described on p. 15-17, 39 and 41 of the revised manuscript (see our reply to the first comment of Reviewer 1 for the additions made to the manuscript).

*Second, the conclusions from modeling relied on fits of individual parameters. However, parameters of models, especially reinforcement learning models, are often difficult to recover reliably due to interdependence (e.g., a low learning rate and high inverse temperature can lead to the same likelihood as a high learning rate and low inverse temperature). In order to believe conclusions from parameter fits, I would first want to see some testing of the reliability of parameter recovery from the model. this can easily be done by simulating data from a known set of parameters, and then attempting to recover those parameters. Alternatively, or better, in addition, confusion matrixes across models can be plotted (that is, simulate data from each of the models, and fit it with all models to test how often the correct model is recovered).*

Reply

We conducted model-recovery analyses and report the results in confusion and inversion matrices (Supplementary Figure 3). Results are summarized on p. 26 of the main text, and the procedure and results are described in detail in Supplementary text 3. In addition, we conducted parameter-recovery analyses for the best-fitting models. Their results are summarized on p. 27 of the main text and described in more detail in Supplementary Text 4 and Supplementary Figures 4-5 (see our reply to the second comment of Reviewer 1 for the additions made to the main text).

*I had several other issues with the modeling: First, I was not sure what is the deviance information criterion (I have never encountered it before) -- how is this different from BIC? Are the results different if using the more standard BIC?*

Reply:

The deviance information criterion (DIC) is a hierarchical modeling generalization of the AIC (Spiegelhalter et al., 2002). It is often used in Bayesian model selection problems where the posterior distributions of the models are obtained by MCMC sampling (as in the present study), because the DIC can be easily calculated from the generated samples, unlike the BIC and AIC. Also note that we pre-registered the use of the DIC. We added some a short explanation of the DIC on p. 17-18:

We compared the models’ performance using the deviance information criterion (DIC), a hierarchical modeling generalization of the AIC which is easily computed in hierarchical Bayesian model-selection problems using Markov chain Monte Carlo (MCMC) sampling [32] (Table 1).

*Second, in Table 1 it was not clear that the difference between the DIC values of model 2B and 1B were significant -- what is the significance of a 1.5% difference between them, aggregated over subjects? It would be more compelling to see statistics, for instance, a t test on the difference in scores between the two models, across participants.*

Reply:

To get a better intuition of the meaning of the DIC differences we transformed the DIC values to model weights (added, in parentheses, in revised Table 1), which can be directly interpreted as conditional probabilities for each model (Wagenmakers & Farrell, 2004), as reported on p. 18:

In addition, we transformed the DIC values to a probability scale (model weights), enabling a more intuitive comparison of the probabilities of each model being the best model, given the data and the set of candidate models [33].

*I would also want to see a measure such as likelihood per trial, which would give some intuition regarding the goodness of fit of the models.* *This is especially important given the very low inverse temperature fits for the choice task -- such a high degree of randomness suggests a poor overall fit, given that the inverse temperature accounts not only for exploration, but also for all other sources of variance unexplained by the model.*

Reply:

We would like to point out that the low inverse-temperature estimates for the choice task do not reflect a poor overall fit, but are the result of the outcome magnitudes used in this task. To illustrate this, consider the softmax function: $P_{1}=1/(1+\exp\left( \beta*\left( Q2-Q1 \right)) \right),$ where $P_{1}$is the probability that option 1 is chosen over option 2, $\beta$ is the inverse temperature, and $Q1$ and $Q2$ are the expected values of options 1 and 2. It can be seen that as $Q2-Q1$ (i.e., delta Q) increases, the inverse temperature associated with the same choice probability decreases. In our task, outcomes were coded on a scale from 0 to 100, and the mean outcomes of the two choice options differed on average 15 points, resulting in delta Q values around 15. This results in much lower inverse temperatures as compared to tasks in which outcomes are coded as 1 vs. 0 such that delta Q values are < 1 (as is typical in tasks with binary outcomes, such as correct vs. incorrect). As an example, when delta Q is 0.5 (a plausible value in tasks with two possible outcomes coded as 1 vs. 0) a 95% probability of choosing the highest-valued option is achieved by an inverse temperature of 6. In contrast, when delta Q is 15 (a plausible value in our task), a 95% probability of choosing the highest-valued option is achieved by an inverse temperature of 0.2. Thus, the inverse temperature estimates in our choice task (Fig 5E, right plot) actually suggest that participants had a rather strong tendency to choose the option with the highest expected value, especially during later trials.

*In Figure 5, the different Y axes in the adult graphs in A (top two) make it hard to read these graphs.*

Reply:

To improve readability, we inserted a break in the y-axes of these two plots, and mention this in the revised legend of Figure 5A:
For visualization purposes, we omitted y-axis values between 40 and 6,550 and between 500 and 23,300, respectively, in the two upper left plots.

*Moreover, for S_1 I was not sure what the units were. Does the amount of initial variance that was fit by the model (in the order of hundreds) make sense?*

Reply:

Yes, those high values for the initial prior variance ($s_{1}^{2}$) make sense. As participants made their first estimate before any outcome had been observed, this estimate could not be based on any prior knowledge; hence was associated with extremely high uncertainty. Such high values of $s_{1}^{2}$ result in initial learning rates that approach 1, which is optimal in our estimation task: as the second estimate could only be based on the first observed outcome, normative behavior entails fully updating (learning rate of 1) one’s second estimate to this first observed outcome. We mention this on p. 21-22 of the revised manuscript:

This makes sense as the first estimate was made before any outcome had been observed, thus could not be based on any prior knowledge (participants basically had to guess on the first trial). Such very high values of $s_{1}^{2}$—relative to the noise variance, $\sigma_{\varepsilon}^{2}$, which was fixed at 1—produce initial learning rates that approach 1 (Equation 3.5 in the Methods), which is optimal in this task.

In addition, in Supplementary text 2, we note that an initial learning rate of 1 is achieved by the Kalman filter when the initial prior variance (relative to the noise variance) is set at a very high value:

Note that this optimal learning rate regime is approached by the Kalman filter when (i) the initial prior variance, relative to the noise variance, is set at a very high value (such that the initial learning rate approaches 1) and (ii) the drift variance, relative to the noise variance, is set at 0 (such that the asymptotic learning rate approaches 0).

*Finally, the modeling results were, for the most part, described without statistical analysis. It is not clear to me that the difference in \sigma^2 between the two age groups was less pronounced (line 387)*

Reply:

Because the Kalman filter is no longer the best-fitting model for the choice task, this result no longer applies. We have rewritten this section, focusing on the new best-fitting choice-task models.

*and that the increase of the inverse temperature was steeper for older adults (line 400).*

Reply:

In the revised manuscript, we tested whether the increase in inverse temperature over trials (trial 20 – trial 1) differed between the two age groups. Importantly, because the best-fitting learning model for the choice task differed between the two age groups (making it difficult to compare the two groups’ estimated softmax parameters), we conducted this analysis twice: First, using the estimated softmax parameters derived from each age group’s respective best-fitting model; and second, using the softmax parameters derived from the same model for both groups. The first analysis revealed no significant difference in the increase in inverse temperature between the two groups (p= .12), reported on p. 23:

Although the increase in inverse temperature was steeper for the adults than the adolescents, the increase in inverse temperature (inverse temperature on trial 20 - inverse temperature on trial 1) did not differ significantly between the two age groups (*t*(58) =1.6, *p* = .12).

However, the second analysis did reveal a significant difference, reported on p. 23-24:

In addition, this analysis revealed that the increase in inverse temperature over trials was significantly larger in the adults than adolescents (*t*(58) =3.1, *p* = .003; Supplementary Figure 1B).

Finally, we explain why we conducted two versions of this analysis on p. 23:

Note that the best-fitting learning models in the choice task differed between the two age groups, which may have affected the group differences in estimated softmax parameters reported above. Therefore, we repeated the group comparisons of $\bar{\theta}$, $\bar{c}$, and inverse temperature, this time using parameter estimates derived from the same model in both age groups: the reinforcement learning/Pearce-Hall hybrid model + dynamic softmax (the winning model for the adults, and shared second best model for the adolescents).

*All of these results would be more convincing with some statistical analysis, rather than just eyeballing the results and describing them. On this last point, statistical analysis was also missing on line 212 -- please compare the learning rates in each age group to the adults if you would like to claim that the regulation of learning rates matures at age 15.*

Reply:

We compared the learning rates in each adolescent age group to the adults, and added the results on p. 11:

To further examine the differences in learning rate within our group of adolescents, we divided the adolescents in three age groups—12/13-year-olds (combined because of the low number of adolescents of each age), 14-year-olds, and 15-year-olds—and compared each of these groups to the adults. These analyses revealed that the 12/13-year-olds used a higher overall learning rate than the adults (main effect of age, *t*(42) = 3.1, *p* = .003), and reduced their learning rate less over time (age x trial-linear and age x trial-quadratic interaction, *t*(42) = 2.1, *p* = .04 and *t*(42) = 3.9, *p* < .001, respectively). The 14-year-olds used a higher overall learning rate than the adults (main effect of age, *t*(41) = 2.4, *p* = .02), but did not differ from the adults in the adjustment of learning rate over time (age x trial-linear and age x trial-quadratic interaction, *t*(41) = 1.7, *p* = .10 and *t*(41) = 1.4, *p* = .17, respectively). Finally, the 15-year-olds did not differ from the adults in either overall learning rate (main effect of age, *t*(41) = .02, *p* = .98) or its adjustment over time (age x trial-linear and age x trial-quadratic interaction, *t*(41) = 1.0, *p* = .32 and *t*(41) = 1.2, *p* = .24, respectively).

*Minor comments:*

*- It was not clear to me until getting to the methods whether participants knew about the block structure. It would be good to make that clear upfront.*

Reply

In the revised manuscript, we make clear that participants knew about the task structure and procedure earlier on, at the beginning of the Results section (p. 8):

Except for the specific means and SDs used, participants were fully informed about the task structure and procedure before starting each task (also see Methods).

*- Figure 3b - why are the bins different for the two age groups?*

Reply:

In this plot, we binned learning rates as a function of certainty ratings, separately for the two age groups. On the x axis, we plotted the mean certainty rating in each bin. Because the certainty ratings were not distributed exactly the same in the adolescents and adults, these values differed between the two age groups.

*- Can the mediation analysis in Figure 6 be done on the reported uncertainty data as well?*

Reply:

Because there are no age effects on reported certainty, we did not look for a mediator of this (non-existing) effect.

*- Will the data and code be made publicly available upon publication?*

Reply:

Yes, data and code will be made available on OSF (https://osf.io/wvqsj/)
