# Supplementary material for: Uncertainty-driven regulation of learning and exploration in adolescents: A computational account
Source: PLoS Comput Biol. 2020 Sep 30;16(9):e1008276. doi: 10.1371/journal.pcbi.1008276 (PMC7549782; doi:10.1371/journal.pcbi.1008276)
Supplement: Attachment — Submitted filename: ReplyToReviewers.docx [file pcbi.1008276.s014.docx]

We thank the reviewers for their remaining comments. Below, the reviewers’ comments are typed in italics, our replies in normal (black) font, and the changes/additions we made in the manuscript in blue.

**Reviewer #1**

*I found very intriguing that the model 2B (asymmetric reinforcement-learning model + dynamic softmax) performed best for the adolescents. I could find the information about the obtained values of alpha(+) and alpha(-). As there is some degree of disagreement in the literature (with Gershman 2015 reporting a negativity bias, Lefebvre 2017 reporting a positivity bias and Van Den Bos 2015 reporting both - as a function of the age) it would be very useful and informative for people in the field to report the results of the positive and negative learning rates of the model 2B as a function of the age group. Other than that, I am satisfied by the revisions.*

Reply:

We report the estimated values of the positive and negative learning rates in Figure 5C (lower panel; posterior distributions of group-level means) and 5E (left panel; posterior medians) and Supplementary Table 1 (posterior medians are around .5 for both learning rates), and describe these results on page 23:
“The winning model for the adolescents had separate learning rates for positive and negative prediction errors, but the group-level means of these two learning rates were highly similar (Fig 5C, lower panel; Fig 5E, left panel). This suggests that some adolescents used higher learning rates for positive than negative prediction errors while others showed the opposite bias (at the individual level, $\alpha_{+}$ was higher than $\alpha_{-}$ for 16 adolescents, and $\alpha_{+}$ was lower than $\alpha_{-}$ for 9 adolescents).”
(this was already present in the previous version of the manuscript)

Thus, at the group level, we found no evidence for a positivity or a negativity bias in the adolescents. The better fit of the asymmetric over the standard RL model in the adolescent group is likely due to individual differences in this bias (i.e., both positivity and negativity biases are present, in different individuals).

**Reviewer #2**

*I have only two remaining concerns, and they are mainly expositional:

The first concern is the unqualified over-interpretation of the meaning of the softmax inverse temperature parameter, as exemplified in the below sentences:

"The degree of choice randomness, or exploration, is often controlled by the inverse-temperature parameter, such that a higher inverse temperature results in a stronger tendency to choose the option with the highest expected value (i.e., less exploration)."

"Importantly, all learning models performed better when combined with a dynamic than a constant softmax function, for both age groups, suggesting that both the adults and adolescents did adjust their degree of exploration over time.”

It is important to remember that the softmax function is not necessarily the way the brain controls exploration — but rather how we link values in our model to probability of choice. This means that all misspecification of a model (…) will be folded into the softmax inverse temperature (…) So while softmax can account for true exploration, we cannot assume that everything it accounts for is exploration.

The first sentence quoted above, in the introduction, conflates what the model does with what the subject does (…)

The second sentence quoted above over-interprets the finding that a dynamic softmax function was a better fit for the data — this could also be due to the model being a worse explanation for the learning phase (…) and a better explanation for the asymptotic phase (…) Indeed, it *could be* that participants are reducing exploration over time, but we cannot know this without testing exploration directly, ideally with a tailored task, or, as you do, with some model-free analysis (…). Given your model-free analysis, this is mainly an expositional point — I have seen so many papers confuse readers who are not versed with model fitting, to think that softmax = exploration. That is just not true.*

Reply:

Thanks for pointing out this important point. In the revised introduction, we changed the first sentence quoted above in such a way that what the model does is not conflated with what the subject does (p. 4):

The probability that the model selects the option with the highest expected valued is often controlled by the *inverse-temperature* parameter, such that a higher inverse temperature increases the likelihood that the model will choose the option with the highest expected value.

Then, we explain that the inverse temperature does not only account for exploration, but for all sources of variance in choice behaviour that cannot be explained by the model (p. 4):

Thus, a lower inverse temperature can account for a more random, or exploratory, choice strategy. However, the inverse temperature also accounts for other sources of variance in choice behaviour that cannot be explained by the model (e.g., due to model misspecifications), which is important to consider when interpreting differences in inverse-temperature estimates between groups or conditions.

In addition, each time we interpret the softmax temperature in terms of exploration, we also mention this other possible interpretation:

p. 17: A higher inverse temperature could reflect a stronger exploitation of the option with the highest expected value, or a better ability of the model to capture participants’ choices.
p. 19-20: all learning models performed better when combined with a dynamic than a constant softmax function, for both age groups, suggesting that both the adults and adolescents did adjust their degree of exploration over time, or that the models’ ability to capture participants’ choices changed as learning progressed.
p. 23: Together, parameters$\theta$ and *c* determine the inverse temperature on each trial (a higher inverse temperature could reflect less exploration, or a better ability of the model to capture the participants’ choices).
p. 23: …reflecting more exploratory choice behaviour in the adolescents, or a worse ability of the model to capture the adolescents’ choices.
p. 23-24: … indicating that participants became less exploratory over time, or that the model was better able to capture participants’ choices during late than early trials.
p. 26: higher values of $\sigma_{\eta}^{2}$ (higher assumed volatility in the estimation task) predicted lower values of $\theta$ (more exploration in the choice task, or a worse ability of the model to capture participants’ choices) and lower values of *c* (a smaller decrease in exploration, or a smaller difference between the model’s ability to capture early and late choices)
p. 42: If $\beta$ is 0, both options are equally likely to be chosen, irrespective of their expected values (reflecting a high degree of choice randomness or exploration, or an inability of the model to capture participants’ choices). As the value of $\beta$ increases, the probability that the stimulus with the highest expected value will be chosen increases (higher degree of exploitation, or better ability of the model to capture participants’ choices).

*My second concern is with this addition: "We compared the learning rates in each adolescent age group to the adults, and added the results on p. 11” — the missing statistical analysis here is the interaction between age and learning rate/learning rate reduction. Differences in p-values in of themselves are not always significant, so the most correct way to do this analysis is to test formally for an interaction with age, as you did for your other analyses. Only if the interaction is significant can we interpret the results you now describe in lines 218-231.*

Reply:

The additional analyses referred to here were added during the first revision in order to test the idea that the regulation of learning rate is matured by the age of 15 (as suggested by Fig. 2C). These specific comparisons were preceded by an analysis in which we included all adolescents and tested for an effect of adolescent age and an age*trial interaction on learning rate (which indicated that learning rate decreased with increasing adolescent age). Thus, we think that the analysis the reviewer asks for is already reported, and we cannot think of another relevant interaction to test for (but if the reviewer has a specific additional analysis in mind, we are happy to add this). However, we realized that the aim of these additional analyses was not stated clearly, which may lead to confusion. Therefore, we changed the sentences that introduce these analyses (p. 11) to:

Figure 2C suggests that learning rates did not differ between the adults and the 15-year-olds, but were elevated in the 12-14-year-olds. To test this idea, we divided the adolescents in three age groups—12/13-year-olds (combined because of the low number of adolescents of each age), 14-year-olds, and 15-year-olds—and compared each of these groups to the adults.

Also note that these were secondary analyses on learning rate, exploring age-related differences within the group of adolescents. In the main analysis on learning rate, which preceded these secondary analyses, we tested for differences between adults and adolescents (without subdividing the adolescents) as well as age group x trial interactions on learning rate (this revealed a main effect of age group and an age group x trial interaction).

*A few more minor comments:

- Although you say in the response letter that you now mention upfront that participants knew when a block changed in the first experiment, I did not find this in the experimental design section (around line 122 and later). Saying that the participants were informed about the task structure in advance does not imply that the block changes were signaled within the task itself. Please add.*

Reply:

In the previous version of our manuscript we said that the start of each new block was signaled to the participants in the methods section (p. 35): ”The start of each new block was clearly indicated, by means of a ‘new-block’ screen, and a different box, with a unique color, was used in each block.”

In the revised manuscript we make this clear earlier on, in the experimental design section (p. 8):

Also, the start of each new block was clearly indicated, by means of a ‘new-block’ screen.

*- It is also not clear from the text or from Figure 1 how many blocks were in the choice task (line 137 suggests more than one — "the means differed 10 or 20 points, in half of the blocks each"), and whether subjects were informed of a block change (that is, are the subjects learning about new options from scratch several times? Or only once?)*

Reply:

We described the number of blocks in the legend of Figure 1, and in the methods section: “Participants completed eight 20-trial blocks of this task”.
We now mention the number of blocks in the revised experimental design section, as well (p. 7): the means differed 10 or 20 points, in four blocks each
Subjects learned about new options from scratch in each block, which was indeed not mentioned explicitly. We added this information in the revised experimental-design section (p. 7-8):
Each block consisted of 20 trials, and participants learned about new choice options from scratch during each block.

*- in line 304 you label the Kalman Filter “Model 2” but you already have another Model 2. You probably also meant to label the Pearce-Hall model “Model 4”.*

Reply:

Thanks for spotting this. We fixed this in the revised manuscript.

*- line 380 — I still wish there were some way to express the model fit in terms of (on average) how likely is each choice under the best fitting model (and each of the others). I find this very intuitive to understand (as we know that for a choice among two options, 50% is chance etc.). I know this is not trivial for MCMC fitting, but on the other hand, you extract*

Reply:

We now computed the probability of each choice under each model, using the posterior medians of the individual-level parameters and the outcomes observed up to that choice, and report the average probabilities (+ SD) in Supplementary Table 2. We also summarize these results in the main text, on p. 20:

For fits to the choice data, we also computed the probability of each choice under each of the models—given the posterior medians of the individual-level parameters and the outcomes observed up to that choice—as an intuitive index of how well the models explain the choice data. The average probability of the observed choices (averaged across trials and participants) under the best fitting model was .78 for the adults, and .65 for the adolescents, consistent with the lower inverse temperature for the adolescents. The probabilities of the observed choices under the other models ranged from .56 to .78 for the adults, and from .54 to .65 for the adolescents (Supplementary Table 2).

*- line 395 "reducing their learning rates over time as a function of their estimation certainty.” — as far as I understand, the models did not model the reported estimation certainty, and did not show that the reduction of learning rate was a function of that. This statement is therefore confusing/misleading.*

Reply:

The models did indeed not model participants’ reported uncertainty. With ‘estimation certainty’ in this sentence we referred to the estimation certainty predicted by the Kalman filter on each trial (s^2^, which in turn determines trial-specific learning rate in this model). We agree that this can be confusing/misleading, and therefore removed this statement from the sentence (p. 19), which now reads: “This suggests that both the adolescents and adults used Bayesian inference to update their estimates.”.

*- the figures as uploaded were extremely grainy and rasterized and I could barely read them.*

Reply:

For this revision, we uploaded our figures to the Preflight Analysis and Conversion Engine (PACE) digital diagnostic tool, to ensure that figures meet PLOS requirements.

Finally, as Reviewer 2 indicated that s/he could not find the link to download the data, we added this on the title page:

**Author note**: Data and modeling code are available at https://osf.io/wvqsj/.
